# Sexually Dimorphic Expression of *eGFP* Transgene in the *Akr1A1* Locus of Mouse Liver Regulated by Sex Hormone-Related Epigenetic Remodeling

**DOI:** 10.1038/srep24023

**Published:** 2016-04-18

**Authors:** Cheng-Wei Lai, Hsiao-Ling Chen, Tung-Chou Tsai, Te-Wei Chu, Shang-Hsun Yang, Kowit-Yu Chong, Chuan-Mu Chen

**Affiliations:** 1 Department of Life Sciences, and Agricultural Biotechnology Center, National Chung Hsing University, Taichung 402, Taiwan; 2 Department of Bioresources, Da-Yeh University, Changhua 515, Taiwan; 3 Department of Physiology, National Cheng Kung University, Tainan 701, Taiwan; 4 Institute of Basic Medical Sciences, National Cheng Kung University, Tainan 701, Taiwan; 5 Department of Medical Biotechnology and Laboratory Science, Chang Gung University, Tao-Yuan 333, Taiwan; 6 Department of Thoracic Medicine, Chang Gung Memorial Hospital at Linkou, Tao-Yuan 333, Taiwan; 7 Rong-Hsing Translational Medicine Center, and iEGG Center, National Chung Hsing University, Taichung 402, Taiwan

## Abstract

Sexually dimorphic gene expression is commonly found in the liver, and many of these genes are linked to different incidences of liver diseases between sexes. However, the mechanism of sexually dimorphic expression is still not fully understood. In this study, a *pCAG-eGFP* transgenic mouse strain with a specific transgene integration site in the *Akr1A1* locus presented male-biased EGFP expression in the liver, and the expression was activated by testosterone during puberty. The integration of the *pCAG-eGFP* transgene altered the epigenetic regulation of the adjacent chromatin, including increased binding of STAT5b, a sexually dimorphic expression regulator, and the transformation of DNA methylation from hypermethylation into male-biased hypomethylation. Through this *de novo* sexually dimorphic expression of the transgene, the *Akr1A1*^*eGFP*^ mouse provides a useful model to study the mechanisms and the dynamic changes of sexually dimorphic gene expression during either development or pathogenesis of the liver.

Sex differences are known to exist in many physiological, developmental and regulatory processes^1^,^2^. In addition to the effects of gene expression from the sex chromosomes, genes with sexually dimorphic expression in both sexes are strongly affected by their epigenetic environment, including DNA methylation^3^,^4^,^5^, histone modification^6^,^7^, functional non-coding RNA regulation^8^,^9^,^10^, and transcription factor (TF) binding^11^. In the liver, more than 1,000 genes have been shown to be expressed in a sexually dimorphic manner^12^, which causes differential metabolism and pharmacokinetics between the sexes and may be involved in liver diseases that show varying incidences between the sexes, such as non-alcoholic fatty liver disease (NAFLD)^13^ and hepatocellular carcinoma (HCC)^14^.

During postnatal liver development, substantial changes in gene expression occur for the first time around the age of weaning^15^. Zonal gene expression, which is involved in metabolic changes such as gluconeogenesis and glutamine synthesis, also forms during this period. The formation of zonal gene expression is due to the coordination of transcriptional activator HNF4α and Wnt/β-catenin signaling in the liver^16^,^17^,^18^. Thereafter, liver gene expression undergoes the next changes during the pubertal period^12^,^19^, when the expression of sexually dimorphic genes is dramatically changed by the influence of sex hormones^20^.

Sex hormones modulate growth hormone (GH) secretion during the critical development periods, including the neonatal and pubertal periods. The synthesis and release of GH are controlled by somatostatin (SS) and GH-releasing hormone (GHRH) neurons in the hypothalamus^21^,^22^. During early neonatal development of the brain, sex hormone exposure permanently changes the number of GHRH neurons and the expression of SS and GHRH and further imprints the differential GH secretion patterns in males and females. During puberty, the increasing sex hormones again stimulate the SS and GHRH neurons and activate the sexually dimorphic secretion patterns of GH^23^, thus activating sexually dimorphic gene expression in the liver.

The differential GH secretion patterns also lead to different epigenetic changes between the livers of males and females^7^. A genome-wide analysis of chromatin accessibility in the liver has identified 850 male-biased and 434 female-biased DNase I-hypersensitive (DHS) regions in male and female mouse genomes, respectively^6^. After the male mice were feminized by continuous GH infusion, the accessibilities of 82% of the male-biased DHS regions, which are enriched with STAT5b binding sites, were decreased^6^. Moreover, the sexually dimorphic genes in the liver also showed sexually dimorphic DNA methylation from the neonatal stage to adulthood^24^.

Sexually dimorphic expression of transgenes has been reported in several transgenic (Tg) animals, which were produced either by pronuclear microinjection^25^,^26^ or viral infection^27^. This phenomenon was usually attributed to position-effect variegation, epigenetic modification and *cis*-upstream regulation^28^,^29^; however, the precise mechanisms remain unclear. In this study, we characterize a special line of Tg mice expressing EGFP, which is driven by the CAG (cytomegalovirus intermediate early enhancer, chicken β-actin promoter and rabbit β-globin intron) promoter^30^. The Tg mouse line contained one copy of the transgene, with a precise integration site in the mouse *Akr1A1* locus, which was analyzed in this study, and showed sexually dimorphic expression of the transgene in the liver after puberty. The results of the current experiments indicate that the timing of sex hormone secretion and the changes in the epigenetic environment (including DNA methylation and STAT5b binding ability) near the integration site are important for the sexual dimorphism of *pCAG-eGFP* transgene expression in the liver. In addition, the *eGFP* Tg mice with sexually dimorphic expression may provide a useful model for studies of the regulation of sexual dimorphism and diseases with sex-specific prevalence in the liver.

## Results

### Characterization of the *pCAG-eGFP* Tg mouse line

**Figure 1:**
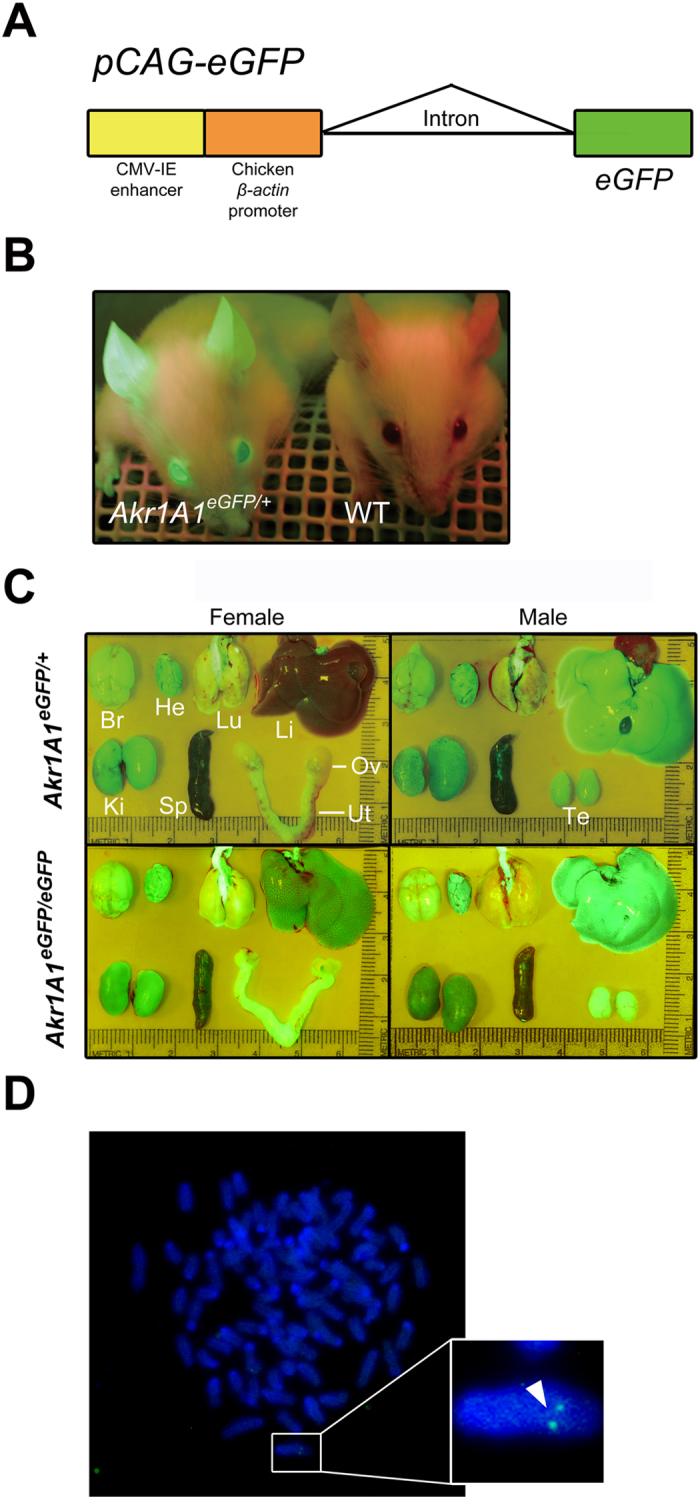
The *pCAG-eGFP* transgenic mice were generated by pronuclear microinjection. (**A**) The structure of the *pCAG-eGFP* transgene. (**B**) Live imaging of the EGFP fluorescence in *Akr1A1*^*eGFP/*+^ and WT mice. (**C**) The *in vivo* EGFP fluorescence imaging of nine organs in the male and female *Akr1A1*^*eGFP/eGFP*^ and *Akr1A1*^*eGFP/*+^ mice. Br: brain, He: heart, Lu: lung, Li: liver, Ke: kidney, Sp: spleen, Ov: ovary, Ut: uterus and Te: testis. (**D**) The chromosome FISH of the sexually dimorphic EGFP-expressing mouse line. The arrowhead shows the transgenic signals in the chromosome.

A 3,253 bp *pCAG-eGFP* expression vector (Fig. 1A) was used to generate Tg mice with strong and ubiquitously expressed EGFP (Fig. 1B). One of the Tg mouse lines showed an interesting expression profile, in which the transgene exhibited sexually dimorphic expression in the liver but only small or no differences between the sexes in other organs, including the brain, heart, lung, kidney, spleen and gonads (Fig. 1C).

**Figure 2:**
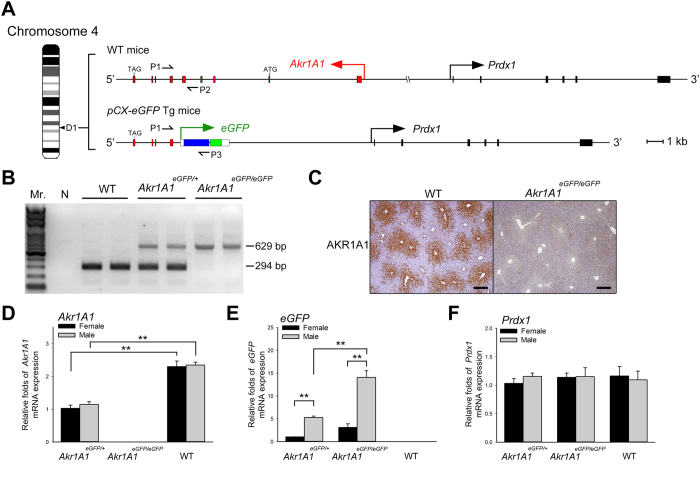
The integration of *pCAG-eGFP* in the Tg mouse line and mRNA expression of *eGFP* and the adjacent genes. (**A**) The proportional map of the *pCAG-eGFP* transgene integration position at the D1 band of mouse chromosome 4. There are 34 kb between exon 1 of *Akr1A1* and the *Prdx1* gene in WT mice, and the integration of the *pCAG-eGFP* transgene caused a ~30-kb genomic deletion, which includes exons 1–5 of the *Akr1A1* gene and the non-coding region between the *Akr1A1* and *Prdx1* genes. The red and black boxes represent the exons of *Akr1A1* and *Prdx1* genes, respectively. The blue and green boxes represent the CAG promoter and *eGFP* of the transgene, respectively. ATG and TAG represent the start and stop codons of the *Akr1A1* gene, respectively. P1, P2 and P3 represent the oligonucleotide primers that were used for the mouse genotyping by PCR. Scale bar: 1 kb. (**B**) The genotyping of the *pCAG-eGFP* mouse line by PCR. The 629 bp and 294 bp amplicons represent the transgenic allele and non-transgenic allele, respectively. (**C**) IHC of the AKR1A1 protein that was expressed in WT but not in *Akr1A1*^*eGFP/eGFP*^ mouse liver. Scale bar: 400 μm. (**D**–**F**) mRNA expression of *Akr1A1, eGFP* and *Prdx1* in the *Akr1A1*^*eGFP/*+^, *Akr1A1*^*eGFP/eGFP*^ and WT mouse livers. The bars show the mean ± s.e.m. of five animals per group (*n* = 5); data were analyzed by a one-way ANOVA with post hoc comparisons using Duncan’s new multiple range test; **represents significant differences (*P* < 0.01).

The integration of the *pCAG-eGFP* transgene in this Tg mouse line was revealed by chromosome fluorescence *in situ* hybridization (FISH) and compatible ends ligation inverse PCR (CELI-PCR). The results showed only one transgene integration site (Fig. 1D), which was located in the region of the *Akr1A1* gene locus that is on the D1 band of mouse chromosome 4 (Fig. 2A). The integration of the transgene caused an approximately 30 kb genomic DNA deletion, including exons 1–5 of the *Akr1A1* gene and the non-coding region between the *Akr1A1* and *Prdx1* genes, which resulted in the knockout of the *Akr1A1* gene and the decreased distance between the transgene and the *Prdx1* gene, which are 10 kb apart (Fig. 2A–D). According to the nomenclature guidelines of the International Committee for Standardized Genetic Nomenclature for Mice, the Tg mice are denoted *Akr1A1*^*Tg(CAG-eGFP)Cmc*^. However, to simplify the nomenclature, the heterozygous (*Akr1A1*^*Tg* (*CAG-eGFP*)*Cmc/*+^) and homozygous (*Akr1A1*^*Tg* (*CAG-eGFP*)*Cmc/Tg* (*CAG-eGFP*)*Cmc*^) Tg mice are indicated by *Akr1A1*^*eGFP/*+^ and *Akr1A1*^*eGFP/eGFP*^, respectively, in the following experiments.

In previous reports, the expression of the transgene was usually affected by the local epigenetic state, or in contrast, the integration of the transgene altered the local epigenetic regulation to affect the adjacent gene expression^28^,^29^. However, analysis of the mRNA expression from the livers of Tg male and female mice showed that sexual dimorphism was only presented in the *eGFP* expression (Fig. 2E) but not in the *Akr1A1* and *Prdx1* expression (Fig. 2D,F). These results suggest that the sexually dimorphic expression of EGFP was controlled by proximal responsive elements near the transgene integration site.

### The male-biased EGFP expression in the Tg mouse liver

**Figure 3:**
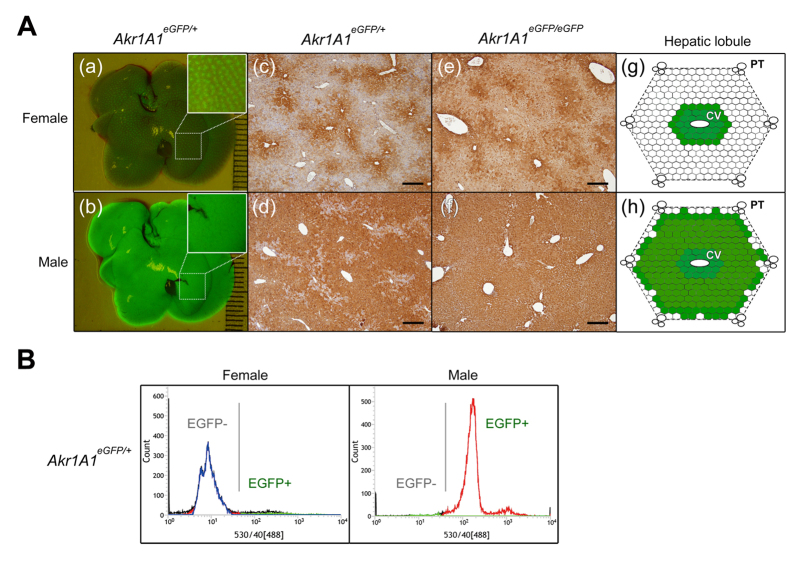
The male-biased EGFP expression in the liver of the *pCAG-eGFP* Tg mouse line. (**A**) The *in vivo* EGFP fluorescence (*Akr1A1*^*eGFP/*+^) (a, b) and IHC of EGFP expression (*Akr1A1*^*eGFP/eGFP*^ and *Akr1A1*^*eGFP/*+^) (c–f) in livers of the male and female Tg mice, and the schematic diagram of EGFP expression in the hepatic lobule (g, h). Scale bar: 400 μm; CV: central vein; PT: portal triad. (**B**) The cell counts of the EGFP+ and EGFP− hepatocytes, which were isolated from male and female *Akr1A1*^*eGFP/*+^ mice, were analyzed by flow cytometry. Similar results were shown in five animals of each group (*n* = 5).

Using *in vivo* fluorescence imaging, livers from male and female mice were easily discriminated; the livers from females displayed green fluorescent spots, and the livers from males displayed a uniform distribution of green fluorescence (Fig. 3A(a,b)). The histological analysis of the EGFP expression in liver sections revealed that EGFP expression in the livers from females was restricted in the hepatocytes around the central vein of the hepatic lobule. In the livers from males, the hepatocytes with EGFP expression also surrounded the central vein, but the expression area was extended to the boundary of the hepatic lobule (Fig. 3A(c–h) and Supplementary Fig. S1). To accurately calculate the proportion of EGFP positive (EGFP+) and EGFP negative (EGFP−) hepatocytes in the livers, the hepatocytes were isolated and analyzed by flow cytometry. The results showed that less than 5% of EGFP+ and over 95% of EGFP+ hepatocytes were found in the livers of female and male mice, respectively (Fig. 3B). Similar results were found by quantitative RT-PCR (Q-PCR) analysis of the EGFP expression (Fig. 2E), which demonstrated male-biased EGFP expression in the Tg mouse liver.

### The EGFP expression changes during development of the Tg mouse liver

**Figure 4:**
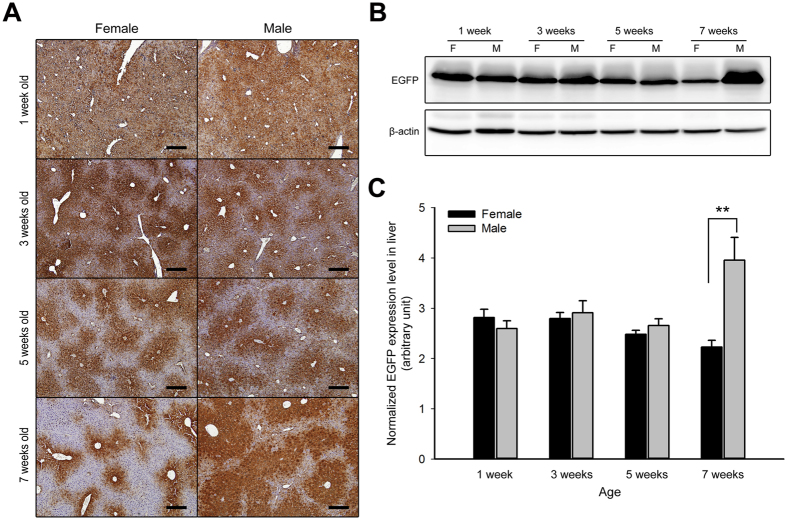
The change in expression of EGFP during the development of the liver in male and female *Akr1A1*^*eGFP/*+^ mice. IHC staining (**A**) and western blot (**B**) analysis of the EGFP protein that was expressed in the livers of 1-, 3-, 5- and 7-week-old male and female Tg mice. Scale bar: 100 μm. (**C**) The quantification of EGFP expression was determined by western blot. β-actin expression was used as an internal control. The bars show the mean ± s.e.m. of five animals per group (*n* = 5); data were analyzed by one-tailed Student’s *t*-test; **P < 0.01 vs. the female group at the age of 7 weeks.

Gene expression in the liver is varied and influenced by metabolic changes and liver function shifts during maturation of the liver. Thus, the EGFP expression in the livers from male and female Tg mice was evaluated at different ages. At 1 week of age, when the mice were still breastfeeding, the EGFP showed neither sex-biased expression nor zonal expression in the hepatic lobule. At the ages of 3 and 5 weeks, when the mice were weaned and at the initial stage of puberty, respectively, zonal expression of EGFP was observed, but there was still no sex-biased expression in the males and females. During late puberty (7 weeks old), sexually dimorphic expression was present (Fig. 4A and Supplementary Fig. S2), and similar results were also shown by *in vivo* EGFP fluorescence imaging (Supplementary Fig. S3). Western blot analysis of EGFP in the Tg mouse livers showed that EGFP expression showed no significant difference between males and females during 1 to 5 weeks of age, but at 7 weeks, the EGFP expression in the livers of males was significantly increased compared to the livers of female mice (*P* = 0.006) (Fig. 4B,C). These results indicate that the sexually dimorphic expression of the *pCAG-eGFP* transgene in the Tg mouse liver was similar to endogenous sexually dimorphic genes^12^, which present male-biased and up-regulated expression after puberty.

### Testosterone administration masculinizes the EGFP expression pattern in the livers of castrated (CX) or ovariectomized (OVX) mice and intact female mice

**Figure 5:**
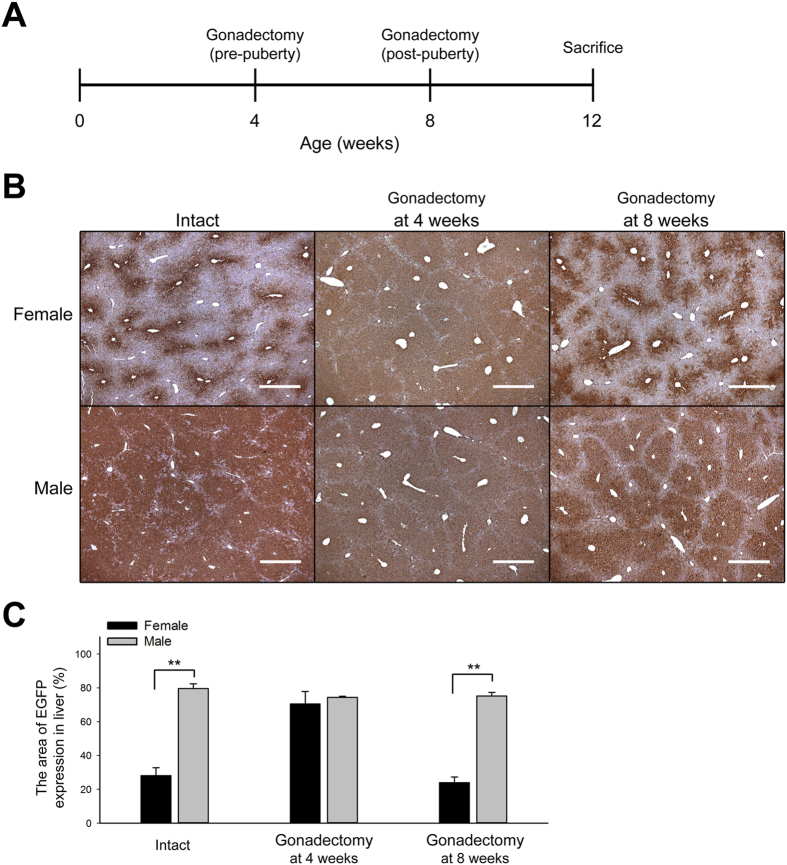
Sex hormones are crucial for the formation of the sexually dimorphic EGFP expression in the *Akr1A1*^*eGFP/*+^ mouse liver during puberty. (**A**) The experimental schedule of Tg mice that underwent gonadectomy at 4 or 8 weeks of age and were sacrificed at 12 weeks of age for IHC analysis. (**B**) IHC of EGFP expression in the livers of intact Tg mice and mice that had undergone gonadectomy at 4 or 8 weeks of age. Scale bar: 500 μm. (**C**) The quantification of the EGFP expression area in IHC sections ([EGFP area (brown color)/total area] × 100%). The bars show the mean ± s.e.m. of five animals per group (*n* = 5); data were analyzed by one-tailed Student’s *t*-test; **P < 0.01 vs. the female group with the same treatment.

To determine whether the sex hormones, which are released during puberty, activated the sexually dimorphic EGFP expression in the mouse livers, we performed gonadectomies in the male and female Tg mice pre-puberty (4 weeks old) and post-puberty (8 weeks old) and then analyzed the EGFP expression at maturity (12 weeks old) (Fig. 5A). The results showed that the sexually dimorphic expression of EGFP was abolished when the operation was performed at 4 weeks old, but there was no change between the intact mice and the mice that underwent gonadectomy at 8 weeks old (Fig. 5B,C). These results indicate that the sex hormones and the timing of their secretion are crucial for the sexually dimorphic expression of EGFP in the Tg mouse livers.

**Figure 6:**
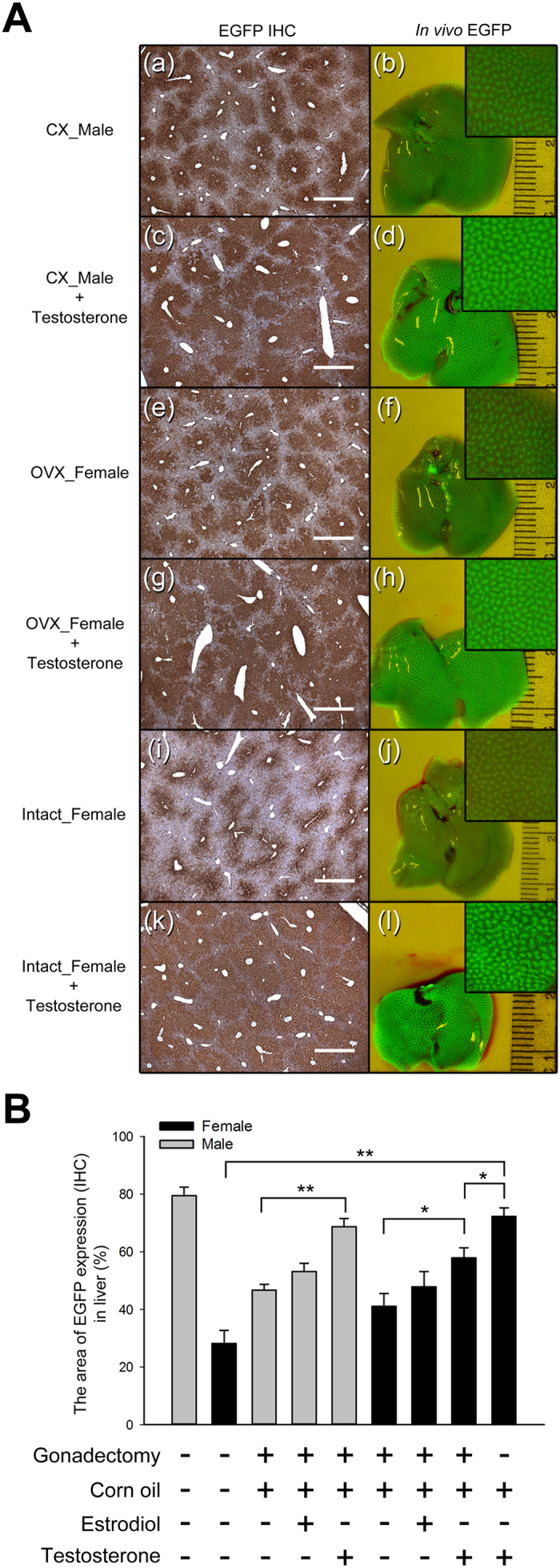
Testosterone masculinizes the expression of EGFP in the *Akr1A1*^*eGFP/*+^ mouse liver. (**A**) The male and female Tg mice that underwent gonadectomy at 4 weeks of age and then received either testosterone (2 mg/kg) or 17β-estradiol (50 μg/kg) administration once a day for 2 weeks; the 8-week-old intact female Tg mice were also treated with the same dose of testosterone. After sex hormone administration, the EGFP expression in the mouse liver was analyzed by IHC and *in vivo* fluorescence imaging. Scale bar: 500 μm. (**B**) The EGFP expression area of the IHC sections, including those from the intact Tg mice and placebo (cone oil) groups, was quantified ([EGFP area (brown color)/total area] × 100%). The bars show the mean ± s.e.m. of six animals per group (*n* = 6); data were analyzed by a one-way ANOVA with post hoc comparisons using Duncan’s new multiple range test; *and **represent significant differences (*P* < 0.05 and *P* < 0.01, respectively).

To further evaluate which sex hormones were involved in the sexually dimorphic EGFP expression, testosterone or 17β-estradiol was administered to mice once a day for 2 weeks (from the age of 4 to 6 weeks) right after the gonadectomy (at 4 weeks old). The expression area of the liver sections and *in vivo* fluorescence imaging were used to evaluate the expression of EGFP. In the CX males and OVX females, testosterone administration significantly increased the EGFP expression area of the livers compared with that of the cone oil control groups (1.5-fold in CX males and 1.4-fold in OVX females, *P* < 10^−4^ and *P* < 0.05, respectively) (Fig. 6A(a–h), B). In addition, in the intact adult female mice (8-week-old) with the same testosterone treatment (from the age of 8 to 10 weeks), the EGFP expression area in the liver sections was 2.5-fold higher than that in the untreated intact females (*P* < 10^−4^) (Fig. 6A(i–l), B). However, there was no significant difference between the 17β-estradiol and cone oil control groups (*P* = 0.11 in CX male; *P* = 0.39 in OVX female). These results indicate that testosterone is responsible for the male-biased EGFP expression in the male livers and is able to masculinize the EGFP expression in the livers of CX, OVX and intact female Tg mice.

### The CpG sites, which are adjacent to the *pCAG-eGFP* integration site, show a sexually dimorphic DNA methylation pattern

**Figure 7:**
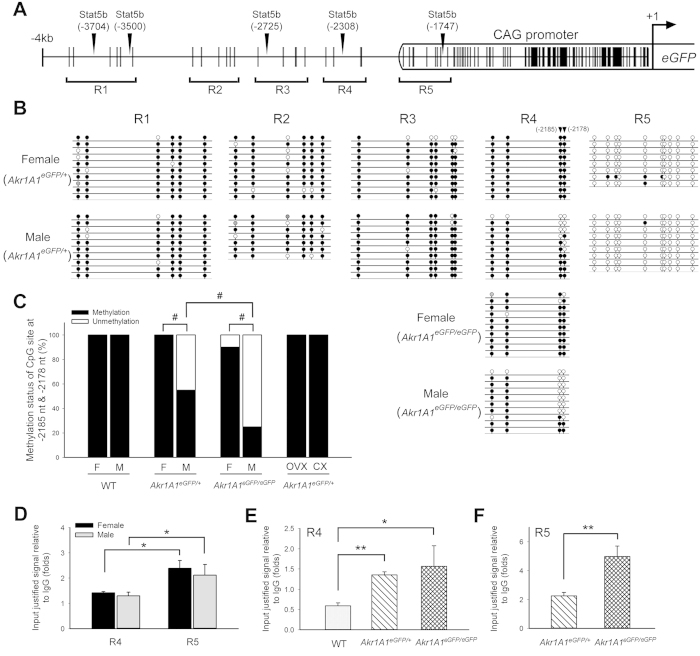
The integration of the *pCAG-eGFP* transgene activated sexually dimorphic DNA methylation and binding of STAT5b in the 5′ adjacent sequence of the transgene. (**A**) A proportional map of the CpG sites (vertical lines) in the 4 kb sequence upstream of the *eGFP* translation start site (+1). The CpG sites are divided into 5 regions, with R1-R4 located in the endogenous mouse genome and R5 located in the CAG promoter of the transgene. The vertical arrowheads represent the locations of the putative STAT5b binding motifs. (**B**) DNA methylation of the R1-R5 regions was analyzed by bisulfite sequencing. Scaled lollipop diagrams show the DNA methylation of the CpG sites in the R1-R5 regions of *Akr1A1*^*eGFP/*+^ mice and the R4 region of *Akr1A1*^*eGFP/eGFP*^ mice. The black and white circles represent methylated and unmethylated CpG sites, respectively, and the gray circles represent indiscriminate sequencing signals. The triangles represent the CpG sites (at −2,178 nt and −2,185 nt) that exhibit sexually dimorphic DNA methylation in the Tg mouse livers. (**C**) The percentage of the CpG site (−2,178 nt and −2,185 nt) methylation in the livers of WT and the Tg mice. F: female; M: male. The data were analyzed by Fisher’s exact test; ^#^*P* < 0.01 between male and female or *Akr1A1*^*eGFP/*+^ and *Akr1A1*^*eGFP/eGFP*^ male mice. (**D**) The STAT5b binding in the R4 and R5 regions in the *Akr1A1*^*eGFP/*+^ mouse livers. The bars show the mean ± s.e.m. of five animals per group (*n* = 5); data were analyzed by a one-way ANOVA with post hoc comparisons using Duncan’s new multiple range test, and *represents significant differences (*P* < 0.05). (**E**,**F**) The STAT5b binding in the R4 and R5 regions in WT, *Akr1A1*^*eGFP/eGFP*^ and *Akr1A1*^*eGFP/*+^ mice. The bars show the mean ± s.e.m. of six animals (*n* = 6), including three males and three females; data were analyzed by one-tailed Student’s *t*-test; *P < 0.05 and **P < 0.01 vs. WT or the *Akr1A1*^*eGFP/*+^ group.

The DNA methylation status of the 4-kb sequence upstream of the *eGFP* translation start site, which includes the CAG promoter of the transgene, was analyzed by bisulfite sequencing in the Tg mouse liver (Fig. 7A). A total of 32 CpG sites, which were divided into 5 regions (R1-R5), were analyzed, and the results showed that the hypermethylated CpG sites were mainly present in the 5′ adjacent regions (R1-R4) and the hypomethylated CpG sites were in the CAG promoter (R5) (Fig. 7B) in both the male and female mice. In addition, two CpG sites at −2,185 nt and −2,178 nt in the R4 region, which are close to the CAG promoter, display sexually dimorphic methylation (Fig. 7B,C). The DNA methylation status of the two CpG sites was 55% and 25% in the *Akr1A1*^*eGFP/*+^ and *Akr1A1*^*eGFP/eGFP*^ male mice, respectively; these values were significantly lower than the approximately 100% methylation status of the CpG sites in both sexes of the wild-type (WT) and in the female Tg mice (*P* < 0.01) (Fig. 7C). Furthermore, the sexually dimorphic DNA methylation was also abolished in the mice that were gonadectomized before puberty and 100% methylation was found in both CX and OVX Tg mouse livers (Fig. 7C). Thus, combining the results of the EGFP expression with the DNA methylation status, the sex hormones, most likely testosterone, influenced the epigenetic environment of the transgene integration region in male mice during puberty and may have consequently activated the sexually dimorphic EGFP expression in the Tg mouse liver.

### The integration of the *pCAG-eGFP* transgene increases the STAT5b binding to the adjacent motif

The STAT5b transcription factor, which is activated in response to the differential GH secretion patterns in males and females, regulates the expression of most sexually dimorphic genes in the liver^31^. Thus, the hypothetical STAT5b binding motifs in the 4 kb sequence, upstream of the *eGFP* translation start site were analyzed by the JASPAR database. There were 5 hypothetical motifs at −1,747 nt, −2,308 nt, −2,725 nt, −3,500 nt and −3,704 nt, with match scores of 6.40, 7.15, 7.10, 7.10 and 7.96, respectively (Fig. 7A). The hypothetical motif at −2,308 nt, which is close to the sexually dimorphic CpG sites (−2,185 nt and −2,178 nt) in the R4 region and the most highly scored motif (−1,747 nt), which is located in the R5 region, were further analyzed for STAT5b binding by chromatin immunoprecipitation (ChIP) assay. The results showed that STAT5b binding in both R4 and R5 was not different between the male and female Tg mouse livers (Fig. 7D). In both male and female *Akr1A1*^*eGFP/*+^ mice, STAT5b binding in R5 was approximately 1.5-fold higher than that in R4 (*P* < 0.05) (Fig. 7D), and this result was identical to that obtained from the match scores of the STAT5b motifs with the JASPAR database. In addition, STAT5b binding in the R4 region of the *Akr1A1*^*eGFP/*+^ and *Akr1A1*^*eGFP/eGFP*^ mice was shown to be 2.3- and 2.6-fold higher than that of the WT group (*P* < 10^−4^ and *P* < 0.05), respectively (Fig. 7E), and the STAT5b binding in the R5 region of the *Akr1A1*^*eGFP/eGFP*^ mice was 2.2-fold higher than that of the *Akr1A1*^*eGFP/*+^ mice (Fig. 7F). These results indicate that the integration of the *pCAG-eGFP* transgene may have increased the chromatin accessibility of the adjacent DNA region and thus increased the STAT5b binding. Together with the sexually dimorphic DNA methylation in the Tg mouse liver, these results show that the sexually dimorphic EGFP expression might be the consequence of epigenetic rearrangement due to the integration of the *pCAG-eGFP* transgene, which thus activated the nearby control elements to regulate the sex-biased EGFP expression.

### Using the *in vivo* EGFP fluorescence imaging to monitor the pathogenesis of methionine- and choline-deficient (MCD) diet-induced NAFLD in the Tg mouse livers

**Figure 8:**
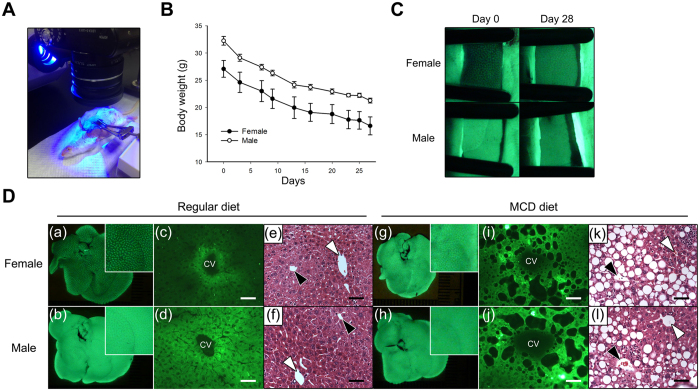
The EGFP expression changes between the livers of the male and female *Akr1A1*^*eGFP/*+^ mice during MCD diet-induced NAFLD. (**A**) The device for EGFP live fluorescence imaging of the Tg mouse liver. (**B**) The body weight change of the Tg mice during MCD diet-induced NAFLD. (**C**) The EGFP live fluorescence imaging of the Tg mouse livers on the 1st and 28th days of MCD diet-induced NAFLD. (**D**) The EGFP expression and morphology of the Tg mouse liver from mice fed a regular or an MCD diet for 4 weeks. The *in vivo* EGFP fluorescence imaging (a, b, g, h); the EGFP fluorescence in the frozen sections (c, d, i, j); H & E staining (e, f, k, l); Scale bar: 50 μm; CV: central vein; the black and white arrowheads represent the location of the central vein and the portal vein, respectively; the body weight is shown as the mean ± s.e.m. of five animals per group (*n* = 5).

The sexually dimorphic EGFP-expressing Tg mice in this study may provide a useful model to study the expression changes of endogenous sexually dimorphic genes. An MCD dietary model, which impairs the VLDL secretion pathway and results in lipid accumulation in the liver, is the most commonly used model to induce NAFLD in rodents. To demonstrate the differential expression changes of the sexually dimorphic EGFP in the livers of male and female mice during NAFLD pathogenesis, the *Akr1A1*^*eGFP/*+^ mice were fed an MCD diet for 4 weeks, and EGFP expression was monitored at day 0 and day 28 by live fluorescence imaging in the same mouse liver (Fig. 8A). The results showed that both the male and female mice lost approximately 35% of their body weight (Fig. 8B) and presented extensive microvesicular steatosis around the central vein of the hepatic lobule after 4 weeks on the MCD diet (Fig. 8D(i–l)), which are significant features of MCD diet-induced NAFLD. EGFP expression was increased in the livers of females at day 28 compared with day 0 in the same mouse, and no obvious changes were observed in the livers of males (Fig. 8C). The results of the *in vivo* EGFP fluorescence imaging showed that the sexual dimorphism of EGFP expression between MCD-fed male and female mice was reduced compared to the Tg mice that were fed a regular diet, and EGFP distribution in the MCD-fed female mice was extended to the hepatic lobules rather than limited to the central vein of the livers as in the regular diet group (Fig. 8D). Accordingly, the change in the sexually dimorphic EGFP fluorescence in the Tg mice livers was followed by pathogenesis of the MCD diet-induced NAFLD, and thus, the *in vivo* EGFP imaging may be useful as a marker for endogenous sexually dimorphic gene expression in the liver.

## Discussion

In this study, we characterized the special expression features of the *pCAG-eGFP* Tg mouse line. The CAG promoter, which was used to promote ubiquitous high-level expression^30^, drives the *eGFP* gene to exhibit sexually dimorphic expression in the mouse liver. The position effect of the transgene has been shown to be a major factor that influences transgene expression in transgenic animals due to the epigenetic regulation of the integration site^28^,^29^. The precise location of the *pCAG-eGFP* transgene in the mouse genome (Fig. 2A) was determined, and the integrating locus (*Akr1A1*) and adjacent gene (*Prdx1*), which are expressed in liver, were not expressed in a sexually dimorphic manner (Fig. 2D,F). Thus, we deduced that the integration of the *pCAG-eGFP* transgene may activate a *de novo* sexually dimorphic regulatory element in the liver genome.

For many endogenous sex-biased genes in the liver, the sexually dimorphic epigenetic environment, including DNA methylation and the binding of transcription factors, has been shown to be capable of influencing the differential gene expression between the sexes in previous studies. Two cytochrome P450 genes, *Cyp2d9* and *Cyp2a4*, are known to display male-biased and female-biased expression in the liver, respectively, and both contain a special CpG site, which is methylated corresponding to the sex-biased expression^5^. Moreover, the male-biased *Slp* gene and its duplicate *C4* gene, which exhibits no sex-biased expression, are distinguished by a male-biased CpG (−112 nt) methylated site that is present in the promoter of the *Slp* gene^32^. These results indicate that methylation of the proximal CpG sites is important and may regulate the sexually dimorphic gene expression in the liver. In the present study, two male-biased demethylated CpG sites (−2,185 nt and −2,178 nt) were found in the 5′ adjacent sequence of the *pCAG-eGFP* transgene in the mouse liver genome (Fig. 7A–C). This male-biased CpG demethylation was correlated with the male-biased EGFP expression in the liver, and notably, the demethylation status in *Akr1A1*^*eGFP/eGFP*^ mice was 2-fold higher than that in *Akr1A1*^*eGFP/*+^ mice, which indicates that these sexually dimorphic CpG sites may only be present in the transgenic allele (Fig. 7C). Similar results in a recent study showed that most of the male-biased DNA demethylated sites occurred exclusively in the livers of males and were close to the adjacent gene (<2 kb). Additionally, the demethylated sites were correlated with the transcription factor (STAT5, BCL6 and RXR) binding sites^24^. Furthermore, cytochrome P450 and many endogenous genes that exhibit sexually dimorphic expression also had sex-dependent DNA methylation sites, which are present in or approximately 2 kb upstream of the transcription start site^4^,^33^,^34^. These results may explain the mRNA expressions in the present study, as the male-biased demethylated CpG sites (−2,185 nt and −2,178 nt) did not influence the expression of the adjacent *Prdx1* gene, which is more than 10 kb away from the CpG sites. Thus, these results strongly suggest that the male-biased EGFP expression in the Tg mouse liver is attributed to the *de novo* sexually dimorphic regulation in the proximal enhancer, which was activated by the transgene integration.

In addition, sexually dimorphic DNA demethylation has been shown to regulate the binding of TFs to their regulatory elements, including the binding of GABP and STAT5b in the male-biased *Cyp2d9* and *Slp* genes, respectively^3^,^5^,^32^. Thus, we analyzed the TF binding in the two STAT5b putative motifs: one that is close to the sexually dimorphic CpG sites (Stat5b^−2308^) and another that is located in the CAG promoter of the transgene (Stat5b^−1747^) (Fig. 7A). However, no sex-biased binding was shown in these two motifs (Fig. 7D), although a previous study, which located chromatin modification and DHS sites in the mouse liver genome, showed that < 50% of the sex-biased genes contain sex-biased chromatin modification sites within 10 kb upstream of the transcription start site^7^, and these chromatin modification patterns may thus influence the binding of TFs. Nevertheless, the STAT5b binding to the motif (Stat5b^−2308^) in *Akr1A1*^*eGFP/*+^ and *Akr1A1*^*eGFP/eGFP*^ mice livers was higher than that in WT mice, and this result further verified the epigenetic rearrangement via the integration of the *pCAG-eGFP* transgene (Fig. 7E).

According to a previous study, 86% of the endogenous sexually dimorphic genes in the liver were observed after puberty^12^, and the male-biased EGFP expression of the Tg mice in the present study was also consistent with this observation (Fig. 4). Furthermore, the results of the mice that had undergone gonadectomy at different developmental stages showed that sex hormone secretion during puberty is crucial for either the male-biased EGFP expression or the sexually dimorphic DNA methylation in the Tg mouse liver (Figs 5 and 7C). The sexually dimorphic expression of the EGFP as well as that of endogenous genes in the liver was abolished or reduced in the mice that had undergone gonadectomy before puberty (Fig. 5 and Supplementary Fig. S4A–C). These results may be due to sexually dimorphic GH secretion patterns (pulsatile and continuous in male and female, respectively), which have been shown to be responsible for most sexually dimorphic gene expression^20^, and were inactivated due to the deficiency in sex hormones during puberty. In a previous study, the mice that underwent hypophysectomy at 8 weeks of age (post-puberty) did not show sex-biased gene expression in the livers^35^; however, the *eGFP* Tg mice that underwent gonadectomy at the same age still exhibited the male-biased EGFP expression (Fig. 5). Although the GH secretion pattern was not detected in the present study, we deduced that the secretion of sex hormones during puberty may imprint the sexually dimorphic GH secretion patterns in the Tg mice. Similar effects have been shown in female mice in which the GH secretion was masculinized by neonatal androgenization^33^, and a recent study showed that the secretion of testosterone at the time of sexual maturity results in long-term stable sex-specific demethylation in livers from males, even when testosterone was deficient after puberty, and this phenomenon was called epigenetic memory^24^. In addition, a previous report showed that re-activation of GH expression in adult CX rats was possible following testosterone replacement, but there was only a slight response to 17β-estradiol^36^. We found similar results here and showed that EGFP expression in the liver could be masculinized by testosterone administration in the CX male and the intact female *eGFP* Tg mice, but no significant changes in the 17β-estradiol groups were observed (Fig. 6). This suggests that testosterone plays an important role in the activation of the male-biased EGFP expression in the Tg mouse liver.

Although transgenes with systemic or liver-specific promoters exhibiting sex-biased expression have been reported in some studies^25^,^26^,^37^, the present study is one of the few to our knowledge that has identified the exact integrated location of the transgene and the interaction between the epigenetics of the adjacent DNA and the sexual dimorphism of the transgene. This *de novo* sexually dimorphic regulation may thus provide a good model for understanding the formation and the mechanism of sexual dimorphism in the liver. In addition, EGFP fluorescence in the liver was able to quantitatively illustrate the sexually dimorphic changes in EGFP in the Tg mouse liver (Figs. 3A and 6). Taking advantage of the *in vivo* EGFP fluorescence imaging, we can detect changes in gene expression during drug metabolism or disease pathogenesis in the liver, e.g., alcohol-induced liver steatosis^38^, NAFLD and HCC, and notably, many sexually dimorphic genes in the liver are also involved in these diseases^39^. Therefore, to verify this idea, we induced NAFLD, a disease that exhibits a sex-specific prevalence in humans^13^,^40^, in the *eGFP* Tg mice by feeding them an MCD diet for 4 weeks. The results showed that the EGFP expression displayed sex-specific changes during the pathogenesis of NAFLD (Fig. 8), and these changes can be used as markers of metabolic changes and endogenous sexually dimorphic gene expression in the livers that differ between sexes.

In conclusion, the *pCAG-eGFP* Tg mouse line exhibits male-biased EGFP expression in the liver, which was attributed to the *de novo* regulatory elements created by the specific integration of the transgene. Like most endogenous genes with sexually dimorphic expression in the liver, EGFP expression was regulated by a sex hormone during puberty and the epigenetic environment of the transgene. This special EGFP transgenic mouse line may provide a live imaging model for studies of differential gene expression and pathogenesis between male and female livers.

## Methods

### Animals

The animals used in the following procedures were approved by the Institutional Animal Care and Use Committee (IACUC No. 103-97) of National Chung Hsing University. The CD-1 mice, which were purchased from the National Laboratory Animal Center (Taipei, Taiwan), were used for Tg mice production. The *pCAG-eGFP* Tg mice were generated by the pronuclear microinjection method^41^,^42^. The original founder mice (F0) were bred with WT mice for at least 2 generations to avoid multiple integration of the transgene in the single Tg mouse line. The procedures were carried out in accordance with the approved guidelines.

### Chromosome fluorescence *in situ* hybridization (FISH)

The transgene integration of the Tg mouse line, which exhibits sexually dimorphic EGFP expression in the liver, was analyzed by chromosome FISH. Briefly, an ear fibroblast cell culture from the Tg mouse line was established, and the cells were incubated with colchicine (0.1 μg/mL) at 37 °C for 2 h and collected by trypsinization. The cells were incubated in hypotonic solution (75 mM KCl) and fixing solution (methanol: acetic acid = 3:1) and then hybridized with a SpectrumGreen-conjugated DNA probe (*pCAG-eGFP* 3-kb fragment) in hybridization buffer (70% formamide and 10% dextran sulfate in 2X saline-sodium citrate (SSC)) at 37 °C for 16 h. After washing, the chromosomes were stained with DAPI (0.5 μg/mL) and examined by fluorescence microscopy^43^.

### Transgene integration site analysis and genotyping

The transgene integration site of the Tg mouse line was revealed by compatible ends ligation inverse PCR (CELI-PCR)^44^. Briefly, the genomic DNA of the Tg mice was digested with *Bgl*II and *Bam*HI and was ligated into circular form. The circular fragments, which contain the 3′ region of the *pCAG-eGFP* transgene, were amplified by inverse PCR. The PCR fragments were cloned and sequenced to reveal the 3′ integration site of the *pCAG-eGFP* transgene. The 5′ integration site was then revealed by genomic PCR with a sense primer that base paired with the 5′ adjacent sequence. For genotyping, the 294 bp DNA fragments were amplified from the WT allele by the P1 and P2 primers (Tm = 58 °C); the 629 bp DNA fragments were amplified from the transgenic allele by the P1 and P3 primers (Tm = 57 °C) (Fig. 2). The PCR primers are shown in Supplementary Table S1.

### Immunohistochemistry (IHC)

The 3 μm paraffin-embedded sections were prepared from fixed livers and used for immunostaining of EGFP and AKR1A1. Briefly, after the sections were deparaffinized and rehydrated, they were incubated in retrieval buffer (10 mM sodium citrate, 0.05% NP-40, pH 6.0) at 100 °C for 30 min. The sections were blocked with horse serum and incubated with polyclonal rabbit anti-GFP (1:2000; GeneTex, Hsinchu, Taiwan) or polyclonal rabbit anti-AKR1A1 (1:500; Sigma-Aldrich, St. Louis, MO, USA) at 4 °C for 16 h. The sections were then incubated with biotinylated secondary antibody for 30 min, and the signal was amplified by the Elite ABC Kit (Vector Laboratories, Burlingame, CA, USA). Finally, the sections were stained with 3,3′ diaminobenzidine (DAB) and counterstained with hematoxylin. All slide images were observed and captured with a Zeiss Axio microscope (Zeiss, Germany)^45^.

### Quantitative RT-PCR (Q-PCR)

Total RNA was isolated from the mouse liver using TRIzol reagent (Invitrogen Co., Grand Island, NY, USA) according to the manufacturer’s instructions^46^. The total RNA (1 μg) was used directly as a template for first-strand cDNA synthesis. Q-PCR was performed using the relative standard curve method with *β-actin* mRNA as a loading control, and the reactions were performed using the GoTaq^®^ qPCR Master Mix (Promega, Madison, WI, USA) and a Roter-Gene^TM^ 6000 instrument (Corbett Life Science, Mortlake, Australia). The Q-PCR primers are shown in Supplementary Table S1.

### Western blot analysis

The total protein from 5 mg of liver was extracted by RIPA buffer. Twenty micrograms of the total protein was then separated by 12% SDS-PAGE and transferred to a polyvinylidene difluoride (PVDF) membrane. The membrane was blocked with 5% BSA and immunoblotted with polyclonal rabbit anti-GFP (1:4000; GeneTex) and monoclonal mouse anti-β-actin (1:500; Novus Biologicals, Littleton, CO, USA) antibodies for 16 h at 4 °C. After washing, the membrane was incubated with the horseradish peroxidase-conjugated secondary antibody for 1 h at 25 °C, and the protein bands were detected and quantified by enhanced chemiluminescence (PerkinElmer, Waltham, MA, USA) and the ImageQuant LAS 4000 mini system (GE Healthcare Biosciences, Pittsburgh, PA, USA).

### Flow cytometry of hepatocytes

*Akr1A1*^*eGFP/*+^ male and female mice were used for hepatocyte isolation. After anesthetization, the mice were perfused first with 30 mL of saline through the inferior vena cava and then 12 mL of 0.8% trypsin. The livers were excised, washed twice with 4 °C phosphate-buffered saline (PBS), minced and passed through a 35 μm mesh. A single cell suspension was examined by microscope, and the cells were analyzed immediately by a BD Influx flow cytometer (Beckman Dickson, Franklin Lakes, NJ, USA) using FSC (cell size) and SSC (cell granularity) parameters, and the most abundant cell population (80–90%), which was considered to be hepatocytes, was selected and analyzed with the channel using 488 nm light excitation and 530 nm emission.

### Gonadectomy of Tg mice at different ages

The male and female *Akr1A1*^*eGFP/*+^ mice were divided into 3 groups: the intact group and the groups that underwent gonadectomy at 4 and 8 weeks (pre- and post-puberty, respectively). During the operation, the mice were anesthetized by 3% isoflurane, and the testes or ovaries were excised from a 0.5 mm incision. After stitching up the wound, the mice fully recovered within 2 weeks. At 12 weeks of age, the mice were sacrificed, and the EGFP expression in mouse liver was analyzed by IHC and quantified by ImageJ^47^.

### Sex hormone administration

The male and female *Akr1A1*^*eGFP/*+^ mice were divided into 4 groups. Groups 1, 2 and 3 underwent gonadectomy during early puberty (4 weeks) and received subcutaneous (S.C.) injections of testosterone (2 mg/kg; Wako Pure Chemical Industries, Japan), 17β-estradiol (50 μg/kg; Wako Pure Chemical Industries) in corn oil or corn oil only, respectively, once a day for 2 weeks. Group 4 consisted of the mature intact female mice (8 weeks old) that received S.C. injections of testosterone once a day for 2 weeks. After the sex hormone administration, the EGFP expression in mouse liver was analyzed by IHC and quantified by ImageJ.

### Bisulfite sequencing analysis

The DNA methylation status of the R1-R5 regions, which are located 4 kb upstream from the *eGFP* translation start site, was analyzed by bisulfite sequencing^48^. Briefly, the genomic DNA of the mouse liver (500 ng) was used for bisulfite treatment by the EZ DNA Methylation™ Kit (Irvine, CA, USA). The oligonucleotide primers for bisulfite PCR in each region are shown in Supplementary Table S1. After the bisulfite PCR, the purified PCR fragments were cloned into the pGEM-T Easy Vector and then sequenced. For each experimental group, the bisulfite PCR fragments from 5 mice were mixed, and 7–10 clones from each region were sequenced. The results are shown as scaled lollipops by the CpGviewer program^49^.

### STAT5b binding motif prediction and chromatin immunoprecipitation (ChIP)

The STAT5b binding motifs in the 4 kb nucleotide sequence upstream of the *eGFP* translation start site in the *pCAG-eGFP* Tg mouse line were analyzed by the JASPAR database. Two predicted STAT5b binding motifs, one that exhibited sexually dimorphic CpG methylation in the R4 region and another that had the highest prediction score in the R5 region, were further analyzed by a SimpleChIP^®^ Plus Enzymatic Chromatin IP kit (Cell Signaling Technology, Danvers, MA, USA), according to the manufacturer’s instructions. The STAT5 antibody (Cell Signaling Technology) was used for the immunoprecipitation, and normal rabbit IgG was used as a negative control. The DNA fragments from the ChIP were analyzed by Q-PCR. The signal of the DNA fragments from the 2% input chromatin was used as a loading control, and the values were calibrated relative to the IgG control. The Q-PCR primers are shown in Supplementary Table S1.

### Methionine- and choline-deficient (MCD) diet-induced NAFLD

The diet of 8-week-old male and female *Akr1A1*^*eGFP/*+^ mice was changed from regular feed to an MCD diet (Research Diets, New Brunswick, NJ, USA) for 4 weeks. The EGFP expression of the same mouse liver on the first (day 0) and the last (day 28) days of the MCD diet period was imaged by *in vivo* 488 nm light excitation, and the mice were weighed every 2–3 days. At the end of the fourth week, the mice were sacrificed following 16 h of fasting. The mice livers were excised and embedded in paraffin for hematoxylin and eosin (H & E) staining or embedded in Tissue-Tek^®^ O.C.T.™ Compound (Sakura Finetek Europe, Alphen aan den Rijn, Netherlands) for *in vivo* EGFP fluorescence imaging.

### Statistical analysis

All data are presented as the mean ± standard error of mean (s.e.m.). Student’s *t*-test and a one-way ANOVA with Duncan’s new multiple range test were used for the comparisons of two and multiple groups, respectively, and the differences in the DNA methylation status were calculated by Fisher’s exact test. A *P*-value < 0.05 was considered significant.

## Additional Information

**How to cite this article**: Lai, C.-W. *et al*. Sexually Dimorphic Expression of *eGFP* Transgene in the *Akr1A1* Locus of Mouse Liver Regulated by Sex Hormone-Related Epigenetic Remodeling. *Sci. Rep*. **6**, 24023; doi: 10.1038/srep24023 (2016).

## Supplementary Material

Supplementary Information
