# Supplementary material for: Sexually Dimorphic Expression of eGFP Transgene in the Akr1A1 Locus of Mouse Liver Regulated by Sex Hormone-Related Epigenetic Remodeling
Source: Sci Rep. 2016 Apr 18;6:24023. doi: 10.1038/srep24023 (PMC4834580; doi:10.1038/srep24023)
Supplement: Supplementary Information [file srep24023-s1.pdf]

Scientific Reports

Supplementary Information

Sexually Dimorphic Expression of *eGFP* Transgene in the *Akr1A1* Locus of Mouse Liver Regulated by Sex Hormone-Related Epigenetic Remodeling

Cheng-Wei Lai, Hsiao-Ling Chen, Tung-Chou Tsai, Te-Wei Chu, Shang-Hsun Yang, Kowit-Yu Chong and Chuan-Mu Chen*

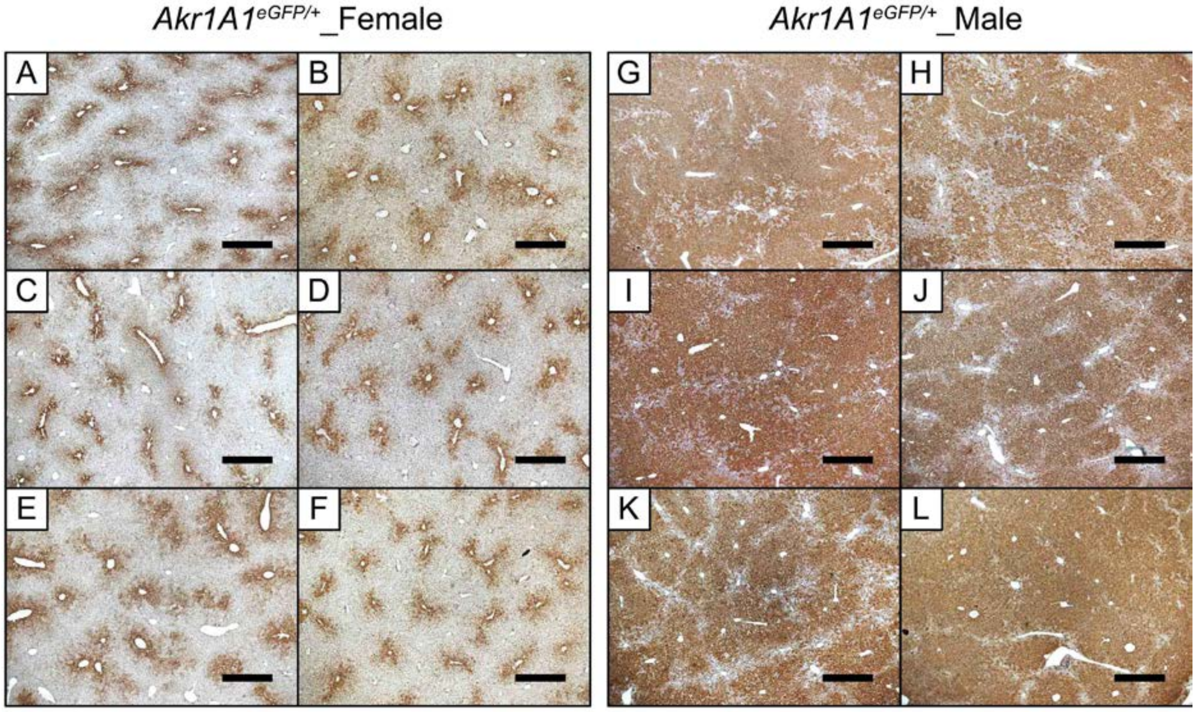

Supplementary Figure S1. The expression of *pCX-eGFP* transgene in the livers of male and female *Akr1A1*$^{eGFP/+}$ mice. The IHC staining of the EGFP expression in the livers from female (A-F) and male (G-L) *Akr1A1*$^{eGFP/+}$ mice (n=6). Scale bar: 500 μm.

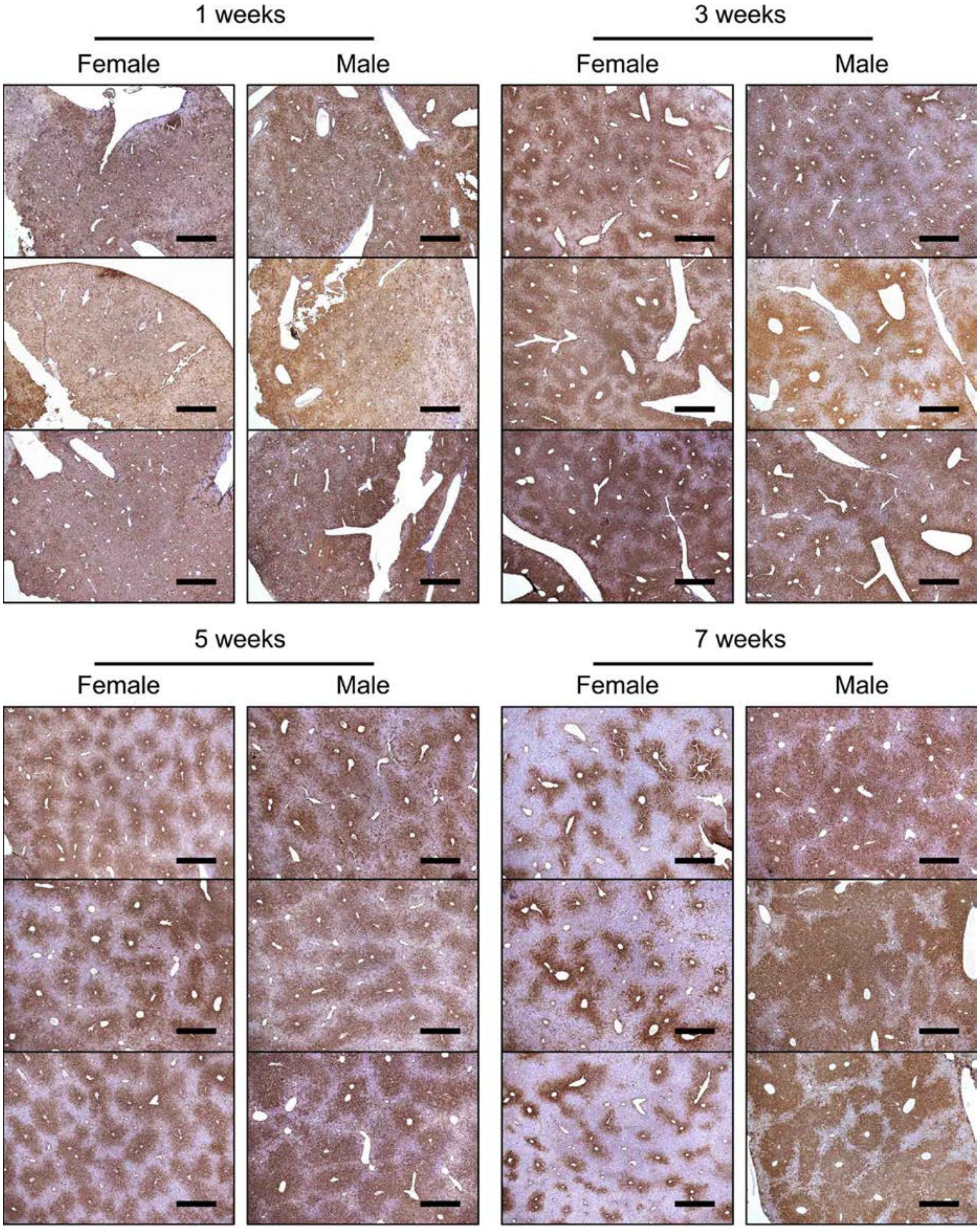

Supplementary Figure S2. The IHC staining of the EGFP expression of the livers of male and female *Akr1A1*$^{eGFP/+}$ mice at 1, 3, 5 and 7 weeks of ages. Scale bar: 500 μm.

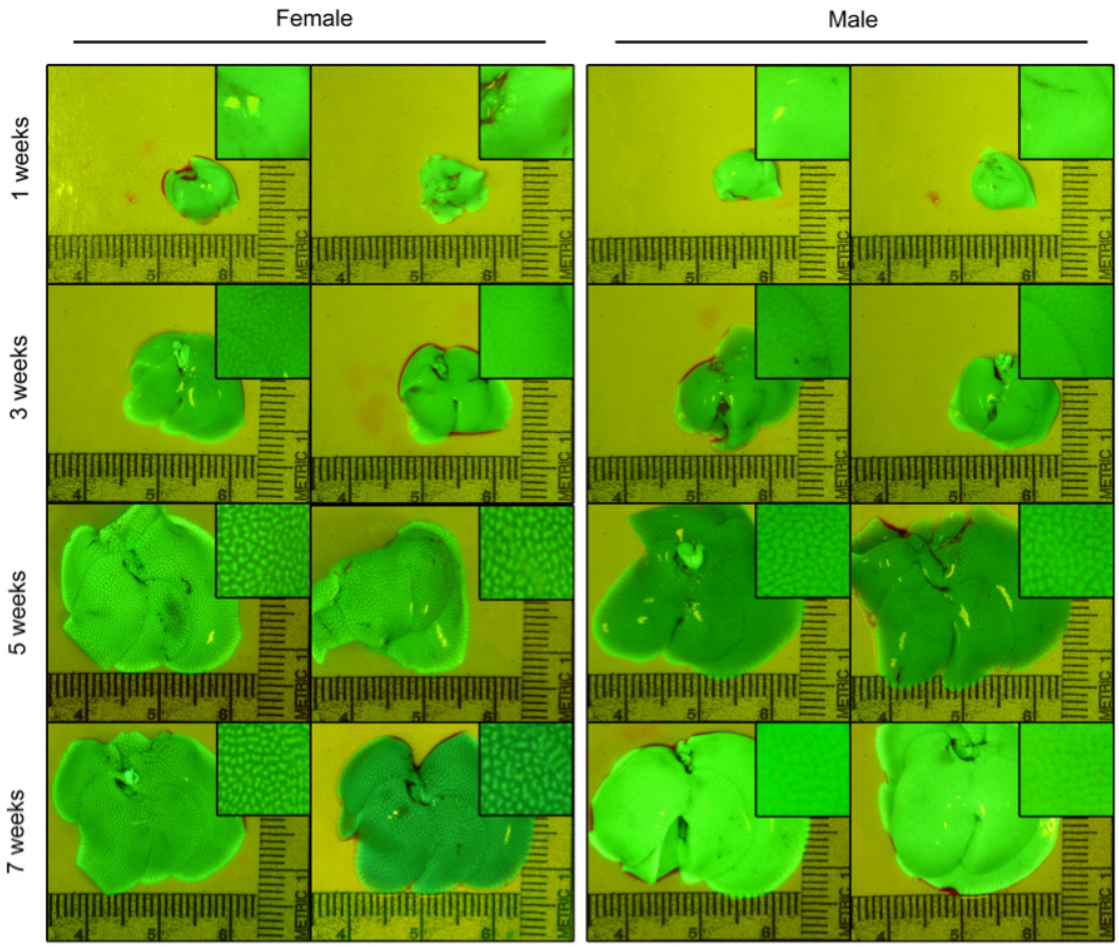

Supplementary Figure S3. The *in vivo* EGFP fluorescence imaging of the livers of male and female *Akr1A1*$^{eGFP/+}$ mice at 1, 3, 5 and 7 weeks of ages.

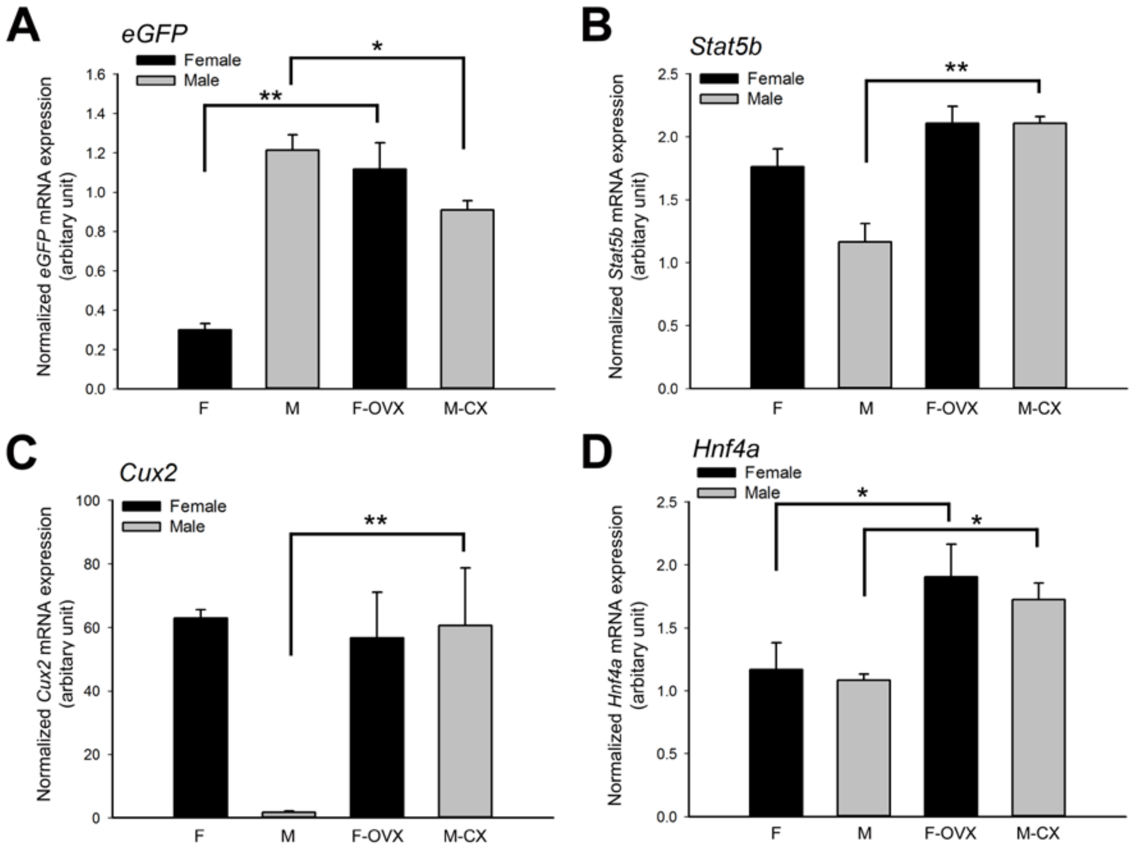

Supplementary Figure S4. The mRNA expression of *eGFP* and genes involved in sexual dimorphism of the livers in intact and castrated (CX) or ovariectomized (OVX) *Akr1A1*$^{eGFP/+}$ mice. The mRNA expression of *eGFP* (**A**), *Stat5b* (**B**), *Cux2* (**C**) and *Hnf4a* (**D**) genes in the livers from different treatments of male and female *Akr1A1*$^{eGFP/+}$ mice. The CX and OVX mice underwent gonadectomy at the age of 4 weeks and were sacrificed at 12 weeks. F: female; M: male; the values are represented as the mean ± s.e.m. of five animals per group (n = 5); data were analyzed by a one-way ANOVA with post hoc comparisons using Duncan's new multiple range test; * and ** represent significant differences ($P < 0.05$ and $P < 0.01$, respectively).

Supplementary Table S1. The list of all PCR primers information used in this study

Experiment	Name	Sequence (5' to 3')		Tm
**Integration site analysis**	5' integration site	(+)<br>(−)	CTGCTAAGAAGCCAGTATTG<br>GTCAATGGAAAGTCCCTATTG	53°C
	3' integration site	(+)<br>(−)	CTGTCCATTCCTTATTCCATAG<br>GCTTCATGATGTCCCCATAAT	56°C
**Genotyping**	P1	(+)	TCCTATCCTCAGCATTAGCTG	See Methods
	P2	(−)	CTTAATGGAGGTGGCAAGTTG	
	P3	(−)	GCTTCATGATGTCCCCATAAT	
**Q-PCR**	*eGFP*	(+)<br>(−)	ACCGGGGTGGTGCCCATCCT<br>TTCACCTCGGCGCGGGTCTT	62°C
	Akr1A1	(+)<br>(−)	CAACTGGAGTATTTGGACCTC<br>GACATCATCAATCTGCCGAC	56°C
	Prdx1	(+)<br>(−)	ACTCTTGTACTCTACTCGTGC<br>GAATGAATCACCAAGTTCTCAG	57°C
	Stat5b	(+)<br>(−)	AGCGTGTTGATAATGGCTTC<br>AAGGGAGATGAAGTGATCTG	53°C
	Cux2	(+)<br>(−)	GTTTGGAGGTGGATGGAGAC<br>GACAGTCTGGATTGGGTGTG	57°C
	Hnf4α	(+)<br>(−)	GTCAGGAACAGTTGTCCATG<br>CCAGAAGACTGCTACCACTG	56°C
	β-actin	(+)<br>(−)	TCAACACCCCAGCCATGTAC<br>AGGATCTTCATGAGGTAGTC	55°C
**Bisulfite sequencing**	R1	(+)<br>(−)	GGTTTTAGTAGGTTTGAGTATATTA<br>CATAAATCATCTTACCTAATCCTC	55°C
	R2	(+)<br>(−)	GTTTTTTTGTATAATTTTGATTGTG<br>ACATATACCACCATTACCTATC	55°C
	R3	(+)<br>(−)	GTGAGTTATTATGTGATTGTTGAG<br>CCACCCTATACTAAAAACTCTTC	56°C
	R4	(+)<br>(−)	GAGTTAGGTATGGATGGTATTTTA<br>CAAACCCTAAATAACAACTAATACT	55°C
	R5	(+)<br>(−)	GTATTAGTTGTTATTTAGGGTTTG<br>CTCCATATATAAACTATAAACTAAT	52°C
**ChIP**	R4-ChIP	(+)<br>(−)	TCACTGGTGAGTTCATTCTC<br>GAGGATAGGATGATAAATGAC	54°C
	R5-ChIP	(+)<br>(−)	AAACAAATAGGGGTTCCGC<br>GCCATTTACCGTAAGTTATG	53°C
